# “Protect, test, vaccinate”: dealing with Covid-19 in outpatient psychiatric care

**DOI:** 10.1192/j.eurpsy.2022.1284

**Published:** 2022-09-01

**Authors:** G. Psota, S. Schuett, B. Vyssoki

**Affiliations:** 1 PSD-Wien , Chefarzt, Wien, Austria; 2 PSD-Wien , Leitung Büro Chefarzt, Wien, Austria; 3 PSD-Wien , Chefarzt Stellvertreter, Wien, Austria

**Keywords:** vaccination, serious mental illnesses, Covid-19

## Abstract

**Introduction:**

Curbing the spread of the coronavirus and stabilizing the overall psychosocial situation requires compliance with preventive measures: “Protect, test, vaccinate”.

**Objectives:**

Population groups with psychosocial problems which are difficult to reach and have a high risk of infection, morbidity and mortality as well as unfavorable help-seeking behavior and generally lower vaccination rates need support.

**Methods:**

In the outpatient psychiatric facilities of the Psychosocial Services in Vienna (PSD-Wien), specific concepts to support “protect, test, vaccinate” were implemented to protect patients and employees. Information about the benefits and risks of vaccination, relieving fears and support in registering and attending vaccination appointments were of special significance.

**Results:**

Analyzes of selected data from 1,319 patients at PSD-Wien show (period: 1st half of 2021) that these measures made it possible to achieve a significantly higher vaccination willingness in people with severe mental illnesses (84 %) than in the general Austrian population (based on the date of examination, currently approximately 60 %). The same applies to vaccination rates: at least 47 % have received a partial vaccination, of which about half have already received both partial vaccinations.

**Conclusions:**

High vaccination willingness and rates as well as the necessary protection (wearing masks, keeping distance, complying with hygiene rules) and regular testing must not be a phenomenon of privileged population groups. Psychosocial support is needed so that the trilogy “Protect, test, vaccinate” becomes possible for everyone, including people with severe mental illnesses. Social psychiatry is not just about mental health, but also about physical health care and prevention.

**Disclosure:**

No significant relationships.

